# Apocrine Hidrocystoma with IgG4 Plasma Cell Infiltration Presenting as Recurrent Chalazion: A Case Report

**DOI:** 10.3390/medicina58070840

**Published:** 2022-06-22

**Authors:** Shang-Yen Wu, Jin-Wei Huang, Yuan-Chieh Lee, Fang-Ling Chang, Ming-Hsun Li, Nancy Chen

**Affiliations:** 1 Department of Ophthalmology, Hualien Tzu Chi Hospital, Buddhist Tzu Chi Medical Foundation, Hualien 970, Taiwan; sun90602@hotmail.com (S.-Y.W.); hgw1120@gmail.com (J.-W.H.); yuanchieh.lee@gmail.com (Y.-C.L.); fangling0103@gmail.com (F.-L.C.); 2 Department of Ophthalmology and Visual Science, Tzu Chi University, Hualien 970, Taiwan; 3 Institute of Medical Sciences, Tzu Chi University, Hualien 970, Taiwan; 4 Department of Pathology, Hualien Tzu Chi Hospital, Buddhist Tzu Chi Medical Foundation, Hualien 970, Taiwan; minghsunli@yahoo.com.tw

**Keywords:** apocrine hidrocystoma, IgG4 plasma cell, chalazion, peri-orbital

## Abstract

Apocrine hidrocystomas are benign cystic tumors resulting from apocrine sweat glands’ proliferation. They typically present as solitary, slow-growing nodules at the head and neck, especially in the periorbital cutaneous region. We present a case of periorbital apocrine hidrocystoma in a 22-year-old woman that was treated as chalazion previously. Besides the hallmark histopathological findings of apocrine hidrocystoma, IgG4 plasma cell infiltration of the cystic wall was also found. The ratio of IgG4-to-IgG-positive plasma cells was high, whereas serum IgG4 was within the standard limit. This is, to date, the only probable IgG4-related ophthalmic disease associated with apocrine hidrocystoma.

## 1. Introduction

Apocrine hidrocystomas are primarily observed in the cutaneous peri-ocular areas, such as eyebrows, upper or lower eyelids, and canthi. They manifest as dome-shaped cystic nodules filled with fluid secreted by the apocrine cells. They rarely present as a lacrimal gland mass [1]. On the contrary, IgG4-related ophthalmic diseases (IgG4-ROD) are orbital lesions frequently involving lacrimal glands, extra-ocular muscles, and infraorbital nerves [2]. The pathological diagnostic features of IgG4-ROD include the presence of lymphoplasmacytic infiltration, a high ratio of IgG4-positive to IgG-positive cells exceeding 40%, and more than 50 IgG4-bearing plasma cells under a high power field of IgG4-ROD [3]. Herein, we present an apocrine hidrocystoma adjacent to the lacrimal gland; it initially appeared as a recurrent chalazion. The histopathological study revealed IgG4-positive plasma infiltrations beneath the apocrine epithelium, and the ratio of IgG4-to-IgG plasma cells was high.

## 2. Case Presentation

**Figure 1:**
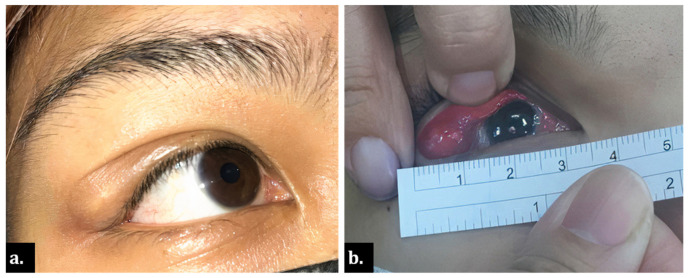
Clinical photograph. (**a**) Yellowish nodule at right upper eyelid appeared as chalazion; (**b**) bulging orbital mass overlaying lacrimal gland, measuring up to 12 mm in diameter.

**Figure 2:**
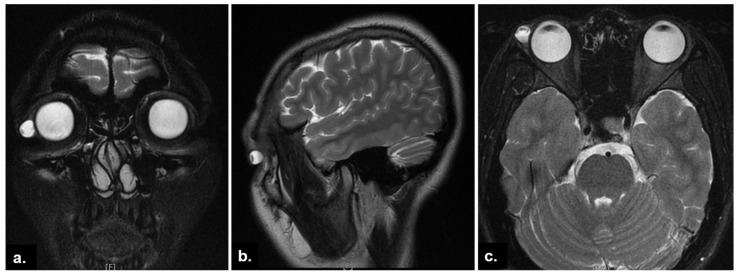
Orbital magnetic resonance images (T2-weighted). (**a**) The coronal image showed a well-defined periocular lesion adjacent to the lacrimal gland; (**b**) sagittal and (**c**) axial images revealed the distinct air-fluid level within the lesion.

A 22-year-old Taiwanese woman was presented for evaluation of an enlarging nodule under her right upper eyelid. The patient described the lesion as tender and causing drooping of her right upper eyelid (Figure 1a). The patient became aware of the lesion two years ago, which had been treated as chalazion with incision and curettage several times at local clinics. She reported no history of ocular trauma or similar conditions in her fellow eye. Her old photographs did not show any corresponding abnormality. Upon ophthalmic examination, an elastic, well-defined, unretractable mass was found under her outer right upper eyelid with overlying conjunctival congestion, deeply extended to the superotemporal orbital rim overlaying the palpebral lacrimal gland area (Figure 1b). Other ophthalmic findings were unremarkable. Orbital magnetic resonance imaging (MRI) demonstrated a space-occupying, well-circumscribed round cystic lesion with a distinct air-fluid level, 8.8 mm × 9.4 mm × 10.7 mm in size, located at the superotemporal orbit, contiguous to the lacrimal gland. The lesion showed hyperintensity in T2-weighted images (Figure 2).

**Figure 3:**
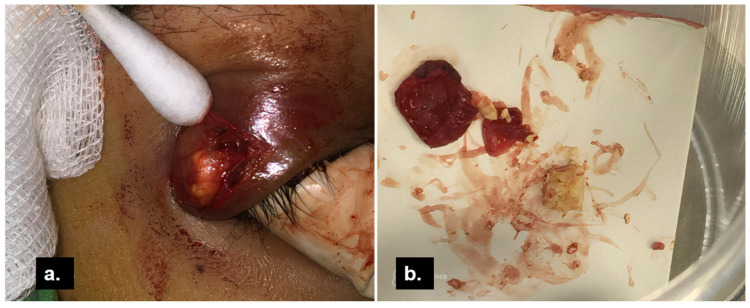
Intraoperative photograph of the cystic lesion. (**a**) After careful dissection of skin crease, orbicularis oculi muscle, and septum, the lesion with fluid and solid components was exposed. (**b**) Although ruptured during dissection, the capsule was removed in whole, and yellowish, caseous contents were expressed from the excised cyst.

**Figure 4:**
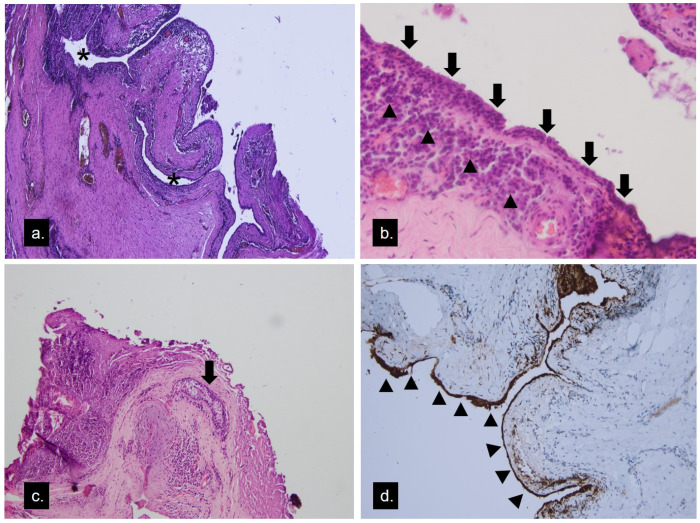
Histopathology of the apocrine hidrocystoma. (**a**) Microscopically, multilocular cystic lesions lined with bilayered cells were seen (asterisk) (hematoxylin–eosin, original magnification ×40). (**b**) Apocrine cells formed the epithelium (arrow), and focal inflamed stroma showed lymphoplasmacytic infiltration (arrowhead) (hematoxylin–eosin, original magnification ×100). (**c**) One lobule of the sebaceous gland (arrow) in the cyst wall (hematoxylin–eosin, original magnification ×40). (**d**) The lining cells showed GCDFP-15 positivity (arrowhead), a marker of apocrine epithelium.

**Figure 5:**
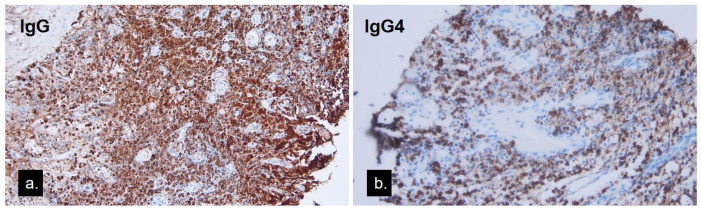
(**a**) Immunohistochemical stain of the lesion using an anti-IgG antibody (**b**) and anti-IgG4 antibody showing the high ratio of IgG4-positive cells against IgG-positive cells exceeding 40%.

Excision of the mass in the whole was done with an external approach through her eyelid crease (Figure 3a). Intraoperative findings identified a well-capsulated mass distinguishable from the lacrimal gland. There was no periosteal involvement. Content extracted from the cyst was greyish-yellow in color and caseous in consistency (Figure 3b). A histopathological study (Figure 4a) showed a cystic lesion lined with bilayered cells. Through magnification, apocrine cells formed the epithelium and focal inflamed stroma with lymphoid hyperplasia (Figure 4b). One lobule of the sebaceous gland was seen in the cyst wall (Figure 4c). In the immunohistochemical study, the lining cells showed gross cystic disease fluid protein-15 (GCDFP-15) positivity (Figure 4d), which was compatible with the apocrine epithelium; hence, the diagnosis of apocrine hidrocystoma was made. The lymphoplasmacytic infiltrations beneath the apocrine epithelium had a high IgG4-to-IgG ratio, exceeding 40% (Figure 5a,b). The cyst contents were sent for microbial examination, which yielded negative findings in acid-fast stain and Streptococcus viridans in bacterial cultures. Oral levofloxacin was prescribed for anti-infection management. Her lab data, including serum IgG and IgG4, were within normal limits. There was no recurrence or other tumefactive lesion found in the orbital or other organs during the three and a half years follow-up.

## 3. Discussion

Hidrocystomas, also known as sudoriferous cysts, are fluid-filled cysts of sweat gland tumors, classified as eccrine and apocrine hidrocystomas based on their origin. Modified apocrine glands include the glands of Moll in the eyelids, the ear-wax-producing ceruminous glands, and the mammary glands. Apocrine hidrocystomas appear as solitary, cystic nodules with varying colors, ranging from flesh-colored to blueish-black [4]. Although an apocrine hidrocystoma is most commonly solitary in its clinical presentation, multiple lesions in the head and neck region have been reported [5]. The pathogenesis of apocrine hidrocystomas is unknown. Some have hypothesized that sequestration of epithelial cells at the embryonic stage may lead to the formation of apocrine hidrocystoma [6]. Meanwhile, epithelial component implantation in a traumatic fashion was also presumed as the etiology [7]. Our patient did not have swelling of the lateral eyelid in her old photographs and underwent several incision and curettage procedures for chalazion treatment that might have introduced epithelium cells into the deeper tissue and, subsequently, formed a noticeable cystic lesion. This is more compatible with the second theory. 

Histopathologically, the cystic wall of apocrine hidrocystoma consists of two layers of epithelium: a luminal layer of apocrine secreting columnar cells over an outer layer of myoepithelial cells. Cytologic features of apocrine glandular cells include “decapitation snouts” at the secretion end of the columnar cells with eosinophilic cytology, GCDFP-15 positivity, and intracellular periodic acid–Schiff positivity [8]. The histopathological study of our case had the bilayer structure with the secreting cell lining and lumen, and the GCDFP-15 stain further proved its apocrine character. However, there were plenty of lymphoplasmacytic infiltrations found in the stroma beneath the secreting epithelium. The immunohistochemical stain further proved them to be IgG4-bearing plasma cells, a feature never reported in apocrine hidrocystomas. 

The incidental finding of IgG4-positive plasma cell infiltration was intriguing. Cyst formation is often preceded by chronic inflammation or trauma. Chronic inflammatory infiltrations, such as in chalazion, are known to contain many plasma cells and lymphocytes. One report found abundant IgG4-bearing plasma cells upon recurrent chalazion tissue biopsy and postulated that it was an IgG4-related disease mimicking recurrent chalazion along with lesions of the trunk, which had the same IgG4 plasma cell infiltrates [9]. Another study found many eosinophils and plasma cells surrounding the lacrimal gland intra-lobular duct cyst associated with vasculitis; however, its immunohistochemical stain of IgG4 of plasma cells showed no significance [10]. IgG4-related disease (IgG4-RD) has been an evolving entity since the 21st century. It is characterized by tumefactive or swelling lesions affecting multiple organs, elevated serum IgG4 exceeding 135 mg/dl, and lymphoplasmacytic infiltration with fibrosis. Additionally, the abundant number of IgG4-positive plasma cells greater than 10 cells per high power field, a high IgG4-to-IgG ratio greater than 40%, along with storiform fibrosis and obliterative phlebitis, were the important histopathological features of IgG4-RD [11]. Organ-specific criteria for ophthalmic components further defined the specific characteristics of IgG4-related ophthalmic disease (IgG4-ROD) as having greater IgG4-positive plasma cells, up to 50 or more per high power field, and less likely to present fibrosis [3]. The most involved sites of IgG4-ROD were well established, including lacrimal glands, extra-ocular muscles, orbital soft tissue, and infraorbital nerves [12]. There were few reports of IgG4-ROD suspected in the isolated lesions of the eyelid and conjunctiva [9,13,14,15]. Nevertheless, there was no previous report of IgG4-ROD speculated in sweat gland cysts or lesions of other folliculosebaceous unit origins like ours. 

The IgG4-ROD was long regarded as a precursor of orbital MALT lymphoma due to similar pathological characteristics in IgG4-positive MALT lymphoma cases [16]. Other conditions, such as rheumatoid arthritis, anti-neutrophil cytoplasmic antibody (ANCA)-associated vasculitis, and atopic dermatitis, might also present infiltration of IgG4-bearing plasma cells [17]. Therefore, the ratio of IgG4-to-IgG was more diagnostic than the absolute numbers of IgG4-bearing cells [11]. Our patient presented with a periorbital swelling nodule, which exhibited infiltration of IgG4-positive plasma cells and a high IgG4-to-IgG ratio greater than 40%; however, her serum IgG4 was not elevated. Hence, the diagnosis was classified as “probable” IgG4-ROD. The manifestation of a high IgG4-to-IgG ratio, as in this apocrine hidrocystoma case, might further expand the spectrum of this inflammatory entity or signify the role of IgG4 plasma cells being a surrogate marker of the inflammatory process. The definite answer needs further investigation for elucidation. 

The hidrocystoma was categorized as one of the entities among the eyelid cystic lesions. These cystic eyelid lesions most commonly originate from the skin and appendages, followed by tarsus, palpebral conjunctiva, and lacrimal glands. In a study of 35 excised eyelid cystic lesions, 16 were epidermoid cysts, 5 intratarsal keratinous cysts, and only 1 apocrine hidrocystoma [18]. Besides the eyelid margin as the most affected site, it appeared on the forehead, external audit canal, post-auricular area, and the orbit [19,20,21]. These cystic lesions are often indistinguishable by clinical pictures, and a biopsy for the pathological survey is mandated for a definite diagnosis. Cutaneous hybrid cysts with components of different origins, such as apocrine hidrocystomas and epidermoid hybrid cysts, were a combination of different types of epithelia found in the folliculosebaceous unit and sweat glands [22,23]. The abrupt transition suggested that these hybrid cysts might arise from junctions rather than metaplasia [22,23]. The fluid content of our study was drained upon rupture of the cyst during dissection; however, the remaining contents retrieved were caseous, resembling the content of a sebaceous cyst. While there were sebaceous lobules contiguous to the cystic wall, it indicated that the contents of the cysts might have sources other than the decapitated apocrine cells. 

Our patient had a tumor in the superotemporal orbit for over two years. According to the patient, the mass had been incised several times for chalazion treatment. In MRI, the cystic mass had a distinct air-fluid level and was palpable at the anterior orbit near the lacrimal gland. The pathological characteristics were consistent with apocrine hidrocystoma; however, there were some unique features. The apocrine cyst might have been the aftermath of the previous surgical procedures that brought skin appendage components into the adjacent tissue. It might have been an inflamed cystic lesion progressively enlarged from the beginning. The IgG4-bearing plasma cell infiltration might result from long-term inflammation, or it was an IgG4-related ophthalmic disease that coincided with the apocrine hidrocystoma. Management of this orbital hidrocystoma remained surgical, with excision of the entire cystic lesion to prevent future recurrence. Despite bacterial infection, presumably caused by normal flora of the conjunctiva, the patient was treated thoroughly and reported no evidence of recurrence. 

## 4. Conclusions

We reported a case of anterior orbital apocrine hidrocystoma with IgG4 plasma cell infiltration. The atypical presentation of this adnexal cystic lesion reminded us of the variable differential diagnosis of peri-orbital diseases, even appearing as a skin-deep chalazion.

## Data Availability

Not applicable.

## References

[1] Mukherjee B., Desai A., Krishnakumar S., Biswas J. (2015). A Giant Apocrine Hidrocystoma Presenting as Lacrimal Gland Mass. Orbit.

[2] McNab A.A., McKelvie P. (2015). IgG4-Related Ophthalmic Disease. Part II: Clinical Aspects. Ophthalmic Plast. Reconstr. Surg..

[3] Goto H., Takahira M., Azumi A. (2015). Diagnostic criteria for IgG4-related ophthalmic disease. JPN J. Ophthalmol..

[4] Hafsi W., Badri T., Shah F. (2022). Apocrine Hidrocystoma. StatPearls.

[5] del Pozo J., García-Silva J., Peña-Penabad C., Fonseca E. (2001). Multiple apocrine hidrocystomas: Treatment with carbon dioxide laser vaporization. J. Dermatol. Treat..

[6] Ssi-Yan-Kai I.C., Pearson A.R. (2012). Recurrent giant orbital apocrine hidrocystoma. Eye.

[7] Mehta A., Rao A., Khanna A. (2008). Sudoriferous cyst of the orbit of adult origin after trauma. Indian J. Ophthalmol..

[8] Barker-Griffith A.E., Streeten B.W., Charles N.C. (2006). Moll gland neoplasms of the eyelid: A clinical and pathological spectrum in 5 cases. Arch. Ophthalmol..

[9] Leivo T., Koskenmies S., Uusitalo M., Tynninen O. (2015). IgG4-related disease mimicking chalazion in the upper eyelid with skin manifestations on the trunk. Int. Ophthalmol..

[10] Mudhar H.S., Currie Z.I., Salvi S.M. (2015). Lacrimal Gland Intra-Lobular Duct Cysts Associated with Focal Vasculitis. Ocul. Oncol. Pathol..

[11] Umehara H., Okazaki K., Kawa S., Takahashi H., Goto H., Matsui S., Ishizaka N., Akamizu T., Sato Y., Kawano M. (2021). The 2020 revised comprehensive diagnostic (RCD) criteria for IgG4-RD. Mod. Rheumatol..

[12] Gan L., Luo X., Fei Y., Peng L., Zhou J., Li J., Lu H., Liu Z., Zhang P., Liu X. (2021). Ophthalmic involvement disparities in clinical characteristics of IgG4-related disease: A retrospective study of 573 patients. BMC Ophthalmol..

[13] Aziz H.A., Villa-Forte A., Plesec T.P., Singh A.D. (2015). Isolated Conjunctival Inflammation Suggestive of IgG4-Related Disease. Ocul. Oncol. Pathol..

[14] Nagai T., Yunoki T., Hayashi A. (2019). A Case of IgG4-Related Bilateral Palpebral Conjunctivitis. Case Rep. Ophthalmol..

[15] Lee H.S., Choi W., Kim G.E., Yoon K.C. (2018). Case of Primary Isolated Subconjunctival IgG4-Related Disease. Cornea.

[16] Oles K., Skladzien J., Szczepanski W., Okon K., Leszczynska J., Bojanowska E., Bartus K., Mika J. (2015). Immunoglobulin G4-related disease (IgG4-RD) in the orbit: Mucosa-associated lymphoid tissue (MALT)-type lymphomas. Med. Sci. Monit..

[17] Strehl J.D., Hartmann A., Agaimy A. (2011). Numerous IgG4-positive plasma cells are ubiquitous in diverse localised non-specific chronic inflammatory conditions and need to be distinguished from IgG4-related systemic disorders. J. Clin. Pathol..

[18] Suimon Y., Kase S., Ishijima K., Kanno-Okada H., Ishida S. (2019). Clinicopathological features of cystic lesions in the eyelid. Biomed. Rep..

[19] Belaldavar B.P., Suranagi V., Kalakuntla M., Raj B., Tiwari A. (2019). Apocrine Hidrocystoma: A Rare Case Report. Indian J. Otolaryngol. Head Neck Surg..

[20] Cavanagh M., Pham M., Nguyen J., Tarbox M. (2021). Postauricular apocrine hidrocystoma: A case and dermoscopy review. Derm. Online J..

[21] Tachibana T., Sasaki T., Wani Y., Naoi Y., Kataoka Y., Nishizaki K., Ando M. (2021). Apocrine Hidrocystoma of the External Auditory Canal in a Child. Otol. Neurotol..

[22] Requena L., Sánchez Yus E. (1991). Follicular hybrid cysts. An expanded spectrum. Am. J. Dermatopathol..

[23] Serra F., Kaya G. (2021). A New Case of Hybrid Epidermoid and Apocrine Cyst. Dermatopathology.

